# Neoadjuvant therapy with camrelizumab plus gemcitabine and cisplatin for patients with muscle‐invasive bladder cancer: A multi‐center, single‐arm, phase 2 study

**DOI:** 10.1002/cam4.5900

**Published:** 2023-04-06

**Authors:** Sujun Han, Zhigang Ji, Junhui Jiang, Xinrong Fan, Qi Ma, Linjun Hu, Wen Zhang, Hao Ping, Jiansong Wang, Wanhai Xu, Benkang Shi, Wei Wang, Haifeng Wang, Honglei Wang, Shouzhen Chen, Hailong Hu, Jianming Guo, Shen Zhang, Shuai Jiang, Quan Zhou, Nianzeng Xing

**Affiliations:** ^1^ Cancer Hospital Chinese Academy of Medical Sciences Beijing China; ^2^ Peking Union Medical College Hospital Beijing China; ^3^ Ningbo First Hospital Ningbo China; ^4^ Cancer Hospital of Huanxing Beijing China; ^5^ Beijing Tongren Hospital Beijing China; ^6^ The Second Affiliated Hospital of Kunming Medical University Kunming China; ^7^ The Fourth Affiliated Hospital of Harbin Medical University Harbin China; ^8^ Qilu Hospital of Shandong University Jinan China; ^9^ The Second Hospital of Tianjin Medical University Tianjin China; ^10^ Zhongshan Hospital Shanghai China

**Keywords:** camrelizumab, muscle‐invasive bladder cancer, neoadjuvant, pathological complete response

## Abstract

**Background:**

Neoadjuvant chemotherapy followed by radical cystectomy (RC) is the standard of care for patients with muscle‐invasive bladder cancer (MIBC). However, treatment outcomes are suboptimal. Camrelizumab, a PD‐1 blockade, has shown benefits in several tumors. This study aimed to investigate the efficacy and safety of neoadjuvant camrelizumab in combination with gemcitabine plus cisplatin (GC) followed by RC for MIBC patients.

**Methods:**

This was a multi‐center, single‐arm study that enrolled MIBC patients with a clinical stage of T2‐4aN0‐1M0, and scheduled for RC. Patients received three 21‐day cycles of camrelizumab 200 mg on day 1, gemcitabine 1000 mg/m^2^ on day 1 and 8, and cisplatin 70 mg/m^2^ on day 2, followed by RC. The primary endpoint was pathologic complete response (pCR, pT0N0).

**Results:**

From May 2020 to July 2021, 43 patients were enrolled and received study medications at nine centers in China. Three of them were deemed ineligible and excluded from efficacy analysis but included in safety analysis. In total 10 patients were unevaluable as they declined RC (two due to adverse events [AEs] and eight due to patient's willingness). Among 30 evaluable patients, 13 patients (43.3%) achieved pCR, and 16 patients (53.3%) achieved pathologic downstaging. No AEs leading to death were observed. The most common AEs were anemia (69.8%), decreased white blood cell count (65.1%), and nausea (65.1%). Immune‐related AEs were all grade 1 or 2. Pathologic response was not correlated with PD‐L1 expression status or tumor mutation burden. Individual genes as a biomarker for pathologic response were not identified.

**Conclusions:**

Neoadjuvant treatment with camrelizumab and GC regimen demonstrated preliminary anti‐tumor activity for MIBC patients with manageable safety profiles. The study met its primary endpoint, and the following randomized trial is ongoing.

## Graphical abstract

In this multicenter, single‐arm study, we investigated the anti‐tumor activity and safety of neoadjuvant camrelizumab plus gemcitabine and cisplatin for MIBC patients. The pCR was 43.3% in evaluable patients. Our study met its primary endpoint, and a randomized trial is currently underway.

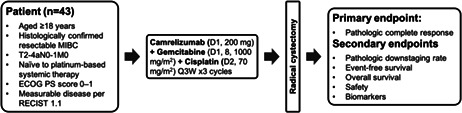

## 1 INTRODUCTION

Bladder cancer is the tenth most commonly diagnosed cancer worldwide, with a higher prevalence in males than females, and Southern Europe, Western Europe, and Northern America being the regions with the highest incidence rates.^1^ Neoadjuvant chemotherapy (NAC) followed by radical cystectomy (RC) is recommended for eligible patients with muscle‐invasive bladder cancer (MIBC).^2^, ^3^ Platinum‐based NACs, including dose‐dense methotrexate, vinblastine, doxorubicin, and cisplatin (ddMVAC) and gemcitabine and cisplatin (GC), are the most commonly used chemotherapy regimens.^4^ Nevertheless, ddMVAC reported more frequent adverse events (AEs).^5^, ^6^

Immune checkpoint inhibitors (ICIs) monotherapy has been approved by the US Food and Drug Administration for treating advanced or metastatic urothelial cancer (UC) in patients who have failed chemotherapy or as first‐line therapy for platinum‐ineligible patients.^7^, ^8^, ^9^, ^10^, ^11^, ^12^, ^13^ In addition, maintenance avelumab has also been approved for the treatment of metastatic UC after chemotherapy.^14^ The efficacy and safety of combined ICI and chemotherapy regimens for advanced or metastatic UC patients have been investigated in two large randomized trials (IMvigor 130 and Keynote 361). Although these trials did not show a significant improvement in overall survival (OS) compared to chemotherapy alone, the combination treatment group showed a numerically higher objective response rate with manageable toxicity.^15^, ^16^

Several trials have demonstrated the anti‐tumor activity of different ICI‐chemotherapy combinations as perioperative treatment for resectable MIBC patients.^17^, ^18^, ^19^, ^20^, ^21^ A phase III trial revealed that camrelizumab (an anti‐programmed death‐1) plus GC regimen had a higher objective response rate and significantly longer progression‐free survival than GC alone in recurrent or metastatic nasopharyngeal carcinoma.^22^ Therefore, we hypothesize that the combination of camrelizumab and GC regimen could potentially be an active regimen for treating MIBC patients in a neoadjuvant setting. In this study, we aimed to investigate the efficacy and safety of neoadjuvant camrelizumab combined with GC followed by RC for MIBC patients, as well as explore the biomarkers for response.

## 2 METHODS

### 2.1 Study design and patients

This investigator‐initiated, multicenter clinical trial evaluated the benefit of neoadjuvant therapy with camrelizumab plus GC in patients with MIBC. The study consisted of two stages: a single‐arm pilot study in the first stage to investigate the combination regimen's efficacy and safety in patients with local advanced resectable MIBC, followed by a randomized controlled study in the second stage to further compare the combination regimen with chemotherapy alone. The second stage would initiate when the first stage met the primary endpoint. Institutional review boards of all participant centers approved the study, and all patients provided informed consent to participate. This study was registered on https://www.chictr.org.cn (ChiCTR2000032359).

Eligible patients were at least 18 years old, diagnosed with histologically confirmed local advanced resectable bladder cancer without metastasis (variant histology allowed without restrictions), naïve to platinum‐based systemic therapy, had at least one measurable bladder lesion according to the RECIST 1.1 criteria, clinical stage of T2‐4aN0‐1M0, scheduled for RC, Eastern Cooperative Oncology Group (ECOG) performance score of 0 or 1, anticipated living longer than 3 months, and had normal hematologic test and sufficient organ function at baseline. Patients with autoimmune or immunodeficiency disease, requiring immunosuppressive therapy in the past 2 weeks, or any other conditions unsuitable for this study, as evaluated by investigators, were excluded.

### 2.2 Procedures and Treatments

During the screening process, all patients underwent a transurethral resection of bladder tumor (TURBT) and underwent imaging (CT or MRI) to assess local and metastatic lesions. Patients were required to have pathologic evidence of muscle invasion in the TURBT specimen prior to enrollment. Tumor tissue and peripheral blood were collected for biomarker analysis.

In the single‐arm stage, patients received neoadjuvant treatment 2 weeks after TURBT. To minimize the time to surgery and potential AEs, three cycles of camrelizumab plus GC were administered. Each cycle consisted of camrelizumab 200 mg intravenously (IV) once on day 1, gemcitabine 1000 mg/m^2^ IV once on day 1 and 8, and cisplatin 70 mg/m^2^ IV once on day 2 every 3 weeks. In the event of treatment‐related adverse events (TRAEs), the related study treatment could be discontinued, while the other two treatments could continue at the discretion of the investigator. RC was performed within 3–4 weeks after the completion of neoadjuvant treatment. As per the standard of care, a standard lymph node dissection was conducted during RC.

### 2.3 Endpoints and assessment

The primary endpoint was pathologic complete response (pCR), which was defined as no viable tumor cells identified in resected tissue (pT0N0). Secondary endpoints included pathologic downstaging (defined as pathologic stage < pT2N0), event‐free survival (EFS, defined as the time from initiation of chemo‐immunotherapy to disease progression, relapse after RC or death from any cause), OS (defined as the time from initiation of chemo‐immunotherapy to death from any cause), and safety. In addition, the study conducted PD‐L1 expression status and whole exoneme sequencing (WES) to explore the potential biomarkers for efficacy. The AEs were reported and graded by National Cancer Institute Common Terminology Criteria for AEs (version 5.0), and toxicities were assessed for 90 days after dosing was complete.

### 2.4 Biomarker analysis

Tissue samples obtained from patients during TURBT and peripheral blood samples were collected for assessing programmed cell death ligand 1 (PD‐L1) expression and WES prior to treatment. PD‐L1 expression was evaluated with the IHC 22C3 pharmaDx (Agilent Technologies). DNA was extracted from TURBT tissue and peripheral blood samples, and invasive tumor content was assessed by pathologists to ensure sufficient tumor cells. DNA was extracted using the DNeasy Blood and Tissue Kit (69,504, QIAGEN). Targeted capture pulldown and exon‐wide libraries were created from native DNA using NadPrep® Hybrid Capture Reagents (for Illumina®, 1,005,101) and NadPrep® DNA Library Preparation Kit (for Illumina®, 1,002,103) E96. Paired‐end sequence data were generated using Illumina NovaSeq machines with an average sequencing depth of 100 × for controls and 200 × for tumors.

The DNA damage response and repair (DDR) gene set, consisting of 276 genes, was built by assembling relevant gene lists, including MSigDB v5.0 (an online catalog of DDR genes from recently published resources), and knowledge‐based curation of information on specific DNA repair pathways or sub‐pathways, as previously reported.^23^ We determined the prevalence of DDR alterations by integrating data on somatic truncating and missense mutations and copy number variants (CNVs). Specifically, GATK 4.0 (Broad Institute), was used to sort and remove polymerase chain reaction (PCR) duplication, and Burrows‐Wheeler aligner was used to align the sequences to the hg19 reference genome. Strelka2 (Illumina) was used to detect SNVs, insertions, and deletions with default parameters. The ANNOVAR (Annotate Variation) was used to annotate possible variant candidates. Somatic CNVs identified by FACETS and recurrently occurring CNVs were detected with GISTIC2.0.

### 2.5 Statistical analysis

Previous studies have shown that patients with MIBC who underwent neoadjuvant GC had a pCR rate of approximately 25%.^24^, ^25^ With the addition of camrelizumab to the GC regimen, it is expected that the pCR rate would increase to 50%. To detect this 25% difference in pCR with a type one error of 10%, a sample size of 29 patients would provide 90% power. If 10 or more patients achieve pCR, the randomized stage of the study will commence. If less than 10 patients achieve pCR, the study will be deemed futile and stopped. However, to account for potential dropouts due to the COVID‐19 pandemic, we plan to recruit at least 40 patients to ensure enough evaluable patients. The single‐arm stage of the study is reported in this article.

All eligible patients who received at least one dose of camrelizumab and underwent cystectomy with post‐operative pathologic assessment was included in the efficacy analysis. The pathologic response will be evaluated by a central pathology committee. Safety analysis will include all eligible patients who received at least one dose of camrelizumab.

Statistical analyses were conducted using SAS 9.4 and R 3.6.0, and a significance level of *p* < 0.05 was used.

## 3 RESULTS

### 3.1 Patients' characteristics

**FIGURE 1:**
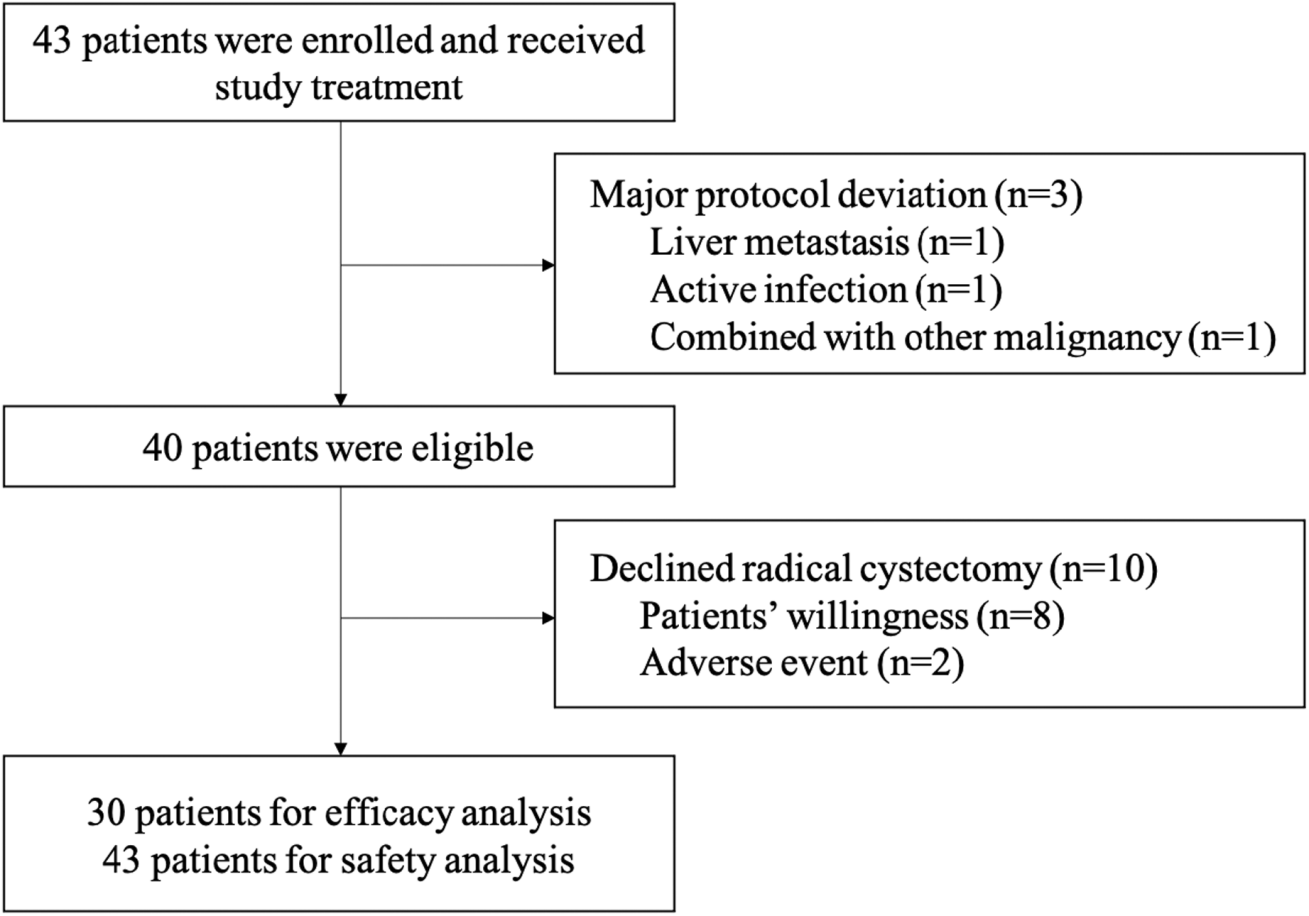
Flowchart of patients.

Forty‐three patients with MIBC were enrolled from May 2020 to July 2021 at nine centers in China, and all received the study drugs. Three of the enrolled patients were found ineligible during the study (one with liver metastasis, one with an active infection, and one with another malignancy) and were excluded from the baseline and efficacy analysis (Figure 1). Ten patients were not evaluable for the primary endpoint since RC was not conducted (two due to AEs, and eight due to patients' willingness). The median age was 67.5 years, and over 75% of the patients were male. Additionally, more than 90% of patients were in clinical stage T2‐3, and over 90% had no prior BCG treatment. The detailed baseline characteristics of patients with or without RC are listed in Table 1.

**TABLE 1:** Baseline characteristics of eligible patients (*n* = 40).

Characteristics	Patients with RC (*n* = 30)	Patients without RC (*n* = 10)
Age (year), median (range)	67.5 (50–81)	67.5 (46–74)
Sex, *n* (%)		
Male	23 (76.7)	9 (90.0)
Female	7 (23.3)	1 (10.0)
TNM stage, *n* (%)		
T2	15 (50.0)	7 (70.0)
T3	12 (40.0)	3 (30.0)
T4a	3 (10.0)	0
N0	27 (90.0)	8 (80.0)
N1	3 (10.0)	2 (20.0)
Histology, *n* (%)		
Pure urothelial	22 (73.3)	6 (60.0)
Major urothelial	8 (26.7)	4 (40.0)
ECOG PS, *n* (%)		
0	12 (40.0)	2 (20.0)
1	17 (56.7)	7 (70.0)
Unknown	1 (3.3)	1 (10.0)
Previous BCG, *n* (%)	2 (6.7)	0
Camrelizumab treatment, *n* (%)		
1 cycle	0 (0.0)	1 (10.0)
2 cycles	1 (3.3)	0 (0.0)
3 cycles	29 (96.7)	9 (90.0)
Cisplatin treatment, *n* (%)		
1 cycle	3 (10.0)	1 (10.0)
2 cycles	3 (10.0)	0 (0.0)
3 cycles	24 (80.0)	9 (90.0)
Gemcitabine treatment, *n* (%)		
1 cycle	2 (6.7)	1 (10.0)
2 cycles	1 (3.3)	0 (0.0)
3 cycles	27 (90.0)	9 (90.0)

*Note*: Three patients found illegible after enrollment were excluded from analyses.

Abbreviations: BCG, Bacillus Calmette‐Guerin; ECOG PS, eastern cooperative oncology group performance status; TNM, tumor, nodes, metastases.

### 3.2 Efficacy

Thirteen out of 30 patients who underwent RC achieved pT0N0M0, resulting in a pCR rate of 43.3% (95% CI, 25.5 to 62.6). At the time of cystectomy, 16 patients achieved < pT2N0M0, with a pathologic downstaging rate of 53.3% (95% CI, 34.3 to 71.7), and none of the patients had disease upstaging (Table 2). The pCR rate and pathologic downstaging rate for all 43 patients were 30.2% (95%CI, 17.2–46.1) and 37.2% (95%CI, 23.0–53.3), respectively. The pCR rates for patients with clinical stages of T2, T3, and T4 at baseline were 46.7% (7/15), 41.7% (5/12), and 33.3% (1/3), respectively. Twenty‐seven patients completed at least two cycles of neoadjuvant treatment and underwent RC. Twenty‐four patients completed all three cycles of neoadjuvant treatment, and 11 (45.8%) patients had a pathological stage of pT0, and three (12.5%) patients had pathological stage < pT2. All patients who underwent RC had negative margins.

**TABLE 2:** Pathologic assessment at the time of radical cystectomy (*n* = 30).

Pathologic stage at RC	*n* (%)
pT0N0M0	13 (43.3, 95% CI, 25.5–62.6)
pTisN0M0	1 (3.3)
pT1N0M0	2 (6.7)
pT2N0M0	5 (16.7)
pT3N0M0	5 (16.7)
pT4N0M0	1 (3.3)
pT1N1M0	1 (3.3)
pT4N1M0	1 (3.3)
pT4N3M0	1 (3.3)

**FIGURE 2:**
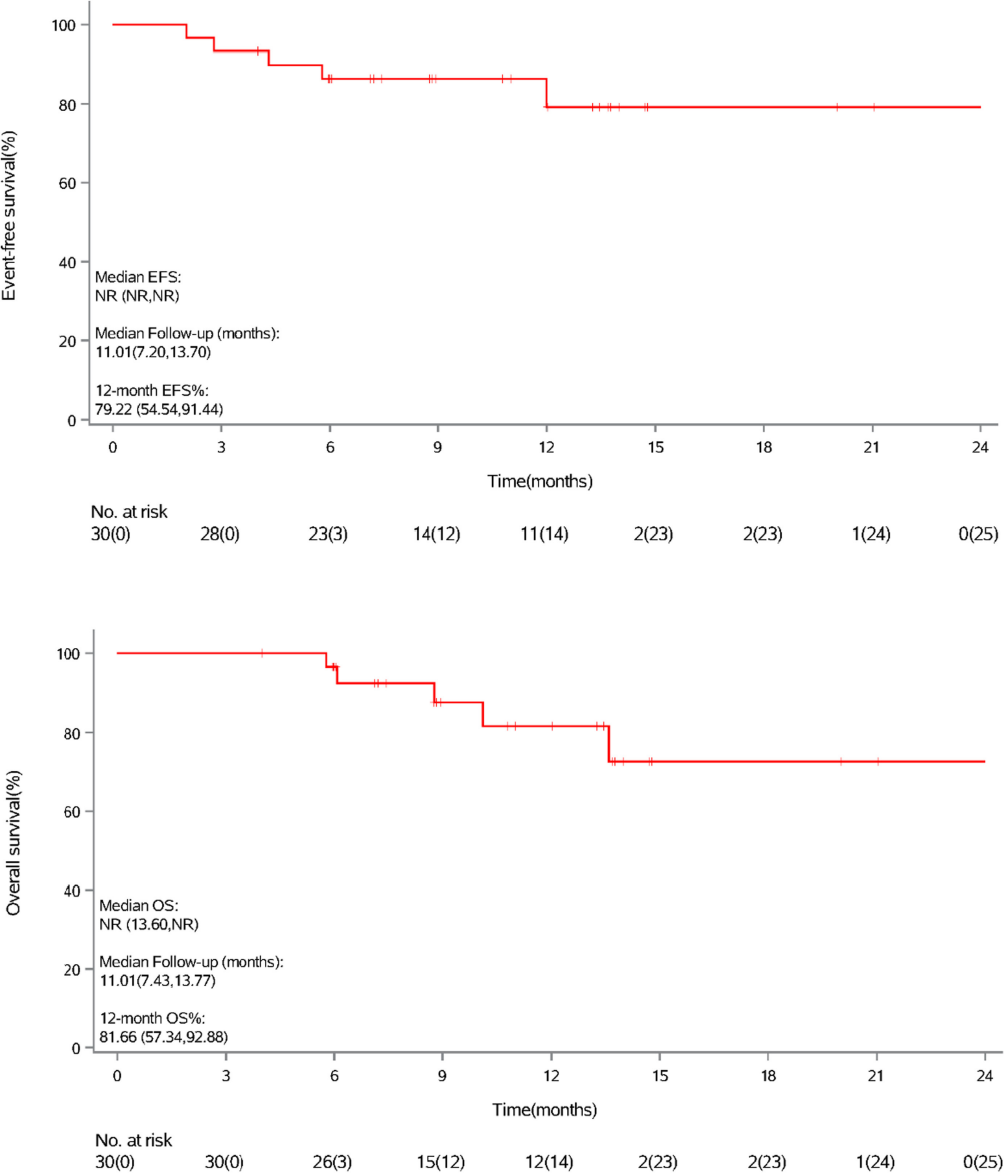
Event‐free survival and overall survival in 30 evaluable patients.

**FIGURE 3:**
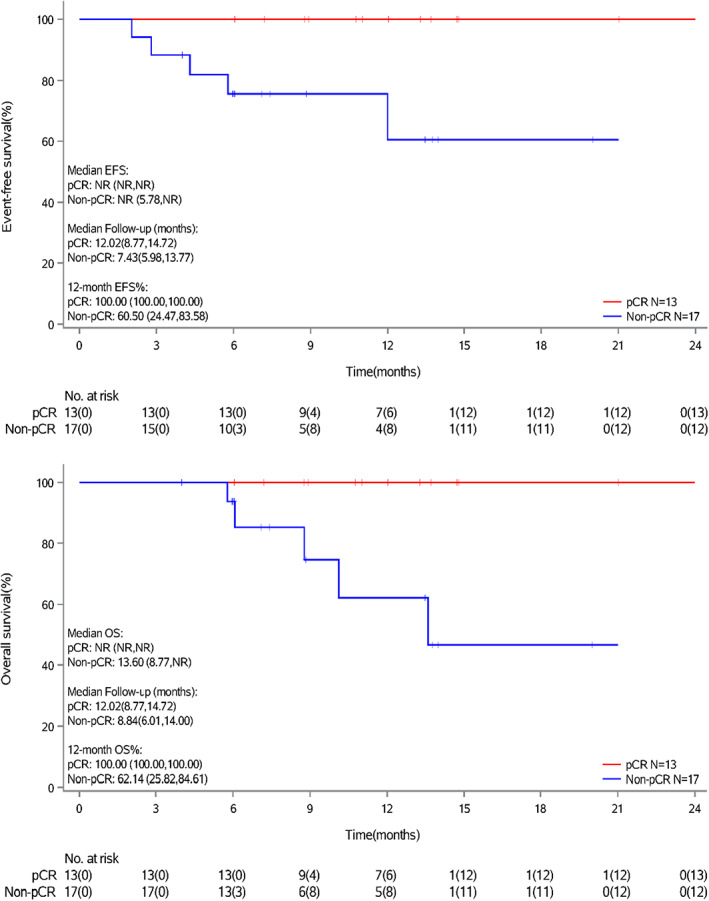
Event‐free survival and overall survival in 30 evaluable patients stratified by pathological response (pathologic complete response [pCR] vs non‐pCR).

After a median follow‐up of 11.0 months (data cut‐off on June 30, 2022), the median EFS and median OS for 30 evaluable patients have not been reached. One‐year EFS and OS rates were 79.2% and 81.7%, respectively (Figure 2). Patients who achieved pCR had better survival than patients without pCR (Figure 3).

Among the 8 patients who did not undergo RC per willingness, the post‐treatment imaging evaluation showed that five patients achieved complete response, one patient achieved partial response, one patient had progressive disease, and one patient was not evaluable. These patients did not undergo repeat cystoscopy.

### 3.3 Adverse events

All 43 enrolled patients received the study drugs and were included for safety analysis. Of these, 36 patients (83.7%) completed three cycles of neoadjuvant therapy. TRAEs of any grade were reported in 95.3% of patients. Grade 3 or 4 TRAEs were reported in 34.9% of patients (Supplementary Table 1). No AE leading to death was observed. Two patients declined RC due to AEs, including one case of cerebral infarction (possibly related to treatment) and one case of urinary frequency (not related to treatment). Eight patients (18.6%) experienced AEs leading to treatment suspension. The most common hematologic AEs were anemia (69.8%), decreased white blood cell count (65.1%), and decreased absolute neutrophil count (55.8%). The most common non‐hematologic AEs were nausea (65.1%), constipation (37.2%), and anorexia (34.9%). All immune‐related adverse events (irAEs) were grade 1 or 2, and none of them required steroids (Table 3).

**TABLE 3:** Treatment‐related adverse events occurring in at least 10% of patients (*n* = 43).

Event, *n* (%)	Grade 1–2	Grade 3	Grade 4	Any grade
Any	26 (60.5)	13 (30.2)	2 (4.7)	41 (95.3)
Hematologic AE				
Anemia	27 (62.8)	3 (7.0)	0	30 (69.8)
WBC decreased	21 (48.8)	7 (16.2)	0	28 (65.1)
ANC decreased	13 (30.2)	11 (25.6)	0	24 (55.8)
ALC decreased	12 (27.9)	1 (2.3)	0	13 (30.2)
Platelet count decrease	6 (14.0)	0	2 (4.7)	8 (18.6)
Nonhematologic AE				
Nausea	27 (62.8)	1 (2.3)	0	28 (65.1)
Constipation	16 (37.2)	0	0	16 (37.2)
Anorexia	15 (34.9)	0	0	15 (34.9)
Rash	11 (25.6)	0	0	11 (25.6)
Vomiting	9 (20.9)	0	0	9 (20.9)
ALT increased	8 (18.6)	0	0	8 (18.6)
Hypoalbuminemia	5 (11.6)	0	0	5 (11.6)
Hyponatremia	5 (11.6)	0	0	5 (11.6)
AST increased	5 (11.6)	0	0	5 (11.6)
Creatinine increased	5 (11.6)	0	0	5 (11.6)
irAE				
Rash	7 (16.3)	0	0	7 (16.3)
Hypothyroidism	2 (11.6)	0	0	2 (11.6)
Urticaria	2 (11.6)	0	0	2 (11.6)
RCCEP	1 (2.3)	0	0	1 (2.3)
Hyperthyroidism	1 (2.3)	0	0	1 (2.3)

Abbreviation: ALC, absolute lymphocyte count; ALT, alanine transaminase; ANC, absolute neutrophil count; AST, Aspartate transaminase; irAE, immune‐related adverse event; RCCEP, reactive cutaneous capillary endothelial proliferation; WBC, white blood cell count.

### 3.4 Biomarkers

Out of the 30 patients evaluated, 28 had undergone baseline WES and PD‐L1 expression examination. No significant correlation between pathologic response and baseline tumor mutation burden (TMB) was observed. However, the median TMB in the pCR group was numerically higher than that in the non‐pCR group (3.75 [range: 0.8, 24.8] versus. 2.45 [range: 0.8, 16.4], *p* = 0.25) (Figure S1A). The combined positive score (CPS) of PD‐L1 expression in both the pCR and non‐pCR groups was similar at baseline (Figure S1B). The top 3 genomic alterations were TP53, FRG1, and TTN. Of the patients in the pCR and non‐pCR groups, 75.0% (9/12) and 68.8% (11/16) had any DDR gene mutation, respectively. The detailed genomic alterations between patients with pCR and non‐pCR were presented in Figure S2. Furthermore, among the seven patients who underwent RC with MDM2 alteration, two had pT0 and two had pT1.

## 4 DISCUSSION

The neoadjuvant treatment of local advanced MIBC patients with three cycles of camrelizumab plus GC demonstrated preliminary anti‐tumor activity in this single‐arm stage. The results of this pilot study met the primary endpoint, and the subsequent randomized stage is currently ongoing to compare camrelizumab plus GC with GC alone as neoadjuvant treatment for patients with local advanced MIBC. These preliminary findings are consistent with other studies that have investigated the use of ICI‐chemotherapy combination regimens as neoadjuvant treatment for MIBC patients. Two pilot studies involving four cycles of pembrolizumab combined with GC demonstrated pCR rates of 36% and 44%, respectively,^18^, ^20^ while a study using atezolizumab plus GC reported a pCR rate of 41% in local advanced MIBC patients.^21^ The short‐course (three cycles) camrelizumab plus GC regimen used in our study provided similar pathologic response compared to the four‐cycle ICI‐chemotherapy regimen. Notably, our study included patients with N1 disease, which is an important consideration when evaluating the efficacy of neoadjuvant therapy for MIBC. Other studies, such as one investigating preoperative four cycles of durvalumab combined with GC and post‐operative ten cycles of durvalumab monotherapy, have also included N1 patients and reported a pCR rate of 30%.^19^ These findings suggest that ICIs in combination with chemotherapy may provide benefits to a wider range of MIBC patients, including those with lymph node involvement, and warrant further investigation in future trials.

The pathologic downstaging rate, as a secondary endpoint, in this study was similar to rates reported in previous studies. Two separate studies that administered four cycles of pembrolizumab combined with GC regimen as neoadjuvant treatment for MIBC patients reported pathological downstaging rates of 56% and 61.1% after resection.^18^, ^20^ Sequential neoadjuvant therapy with lead‐in atezolizumab, four cycles of atezolizumab plus GC and atezolizumab in MIBC patients showed a pathologic downstaging rate of 69%.^21^

The optimal timing and duration of neoadjuvant therapy for MIBC remain topics of ongoing debate. Previous studies have suggested that delaying cystectomy after neoadjuvant therapy may negatively impact patients' outcomes.^26^ Short‐course neoadjuvant ICI‐chemotherapy may reduce the time to RC for local advanced MIBC patients and may be associated with reduced toxicities and lower treatment costs. A recent retrospective study involving 693 patients suggested that three cycles of NAC showed similar pathologic response rates and short‐term survival outcomes compared to four cycles.^27^ Our study also showed that compared with four cycles of ICI‐chemotherapy neoadjuvant treatment, three cycles of ICI‐chemotherapy provided similar pCR rates and pathologic downstaging rates for MIBC patients. Further prospective studies are warranted to confirm these findings.

Patients who achieved pCR in this study had a more favorable 1‐year survival status compared to those who did not achieve pCR. Previous studies have also shown that patients who responded well to neoadjuvant ICI‐chemotherapy had significantly better recurrence‐free survival compared to non‐responders.^20^, ^21^ In patients with local advanced MIBC who received NAC, those in the pCR group had better survival outcomes compared to those in the non‐pCR group.^28^ A longer follow‐up period for this study may potentially identify the association between pathologic response and survival outcomes.

RC is the standard treatment for MIBC. However, some patients may not be eligible for surgery or may prefer to avoid RC due to concerns about quality of life. Therefore, bladder preservation therapies have emerged as an alternative treatment method.^29^ In the phase II HCRN GU 16–257 study, 31 out of 64 MIBC patients who completed TURBT and four cycles of GC plus nivolumab achieved a clinical complete response and did not undergo cystectomy.^30^ In the RETAIN BLADDER study, 26 MIBC patients who achieved a complete response after NAC and had mutations in ATM, ERCC2, FANCC, or RB1 initiated active surveillance without cystectomy.^31^ In our study, eight patients declined RC due to their willingness, including seven patients who were satisfied with the efficacy of NAC and one patient who was concerned about quality of life. Among them, five patients achieved a complete response and one patient achieved a partial response. It appears that bladder preservation may be possible in patients who have a clinical response to neoadjuvant therapy, although further studies are warranted.

The hematologic toxicity profile of this study was similar to that of other MIBC patients treated with neoadjuvant ICI‐chemotherapy.^20^, ^21^ The most common grade 3/4 treatment‐related hematologic AEs were decreased absolute neutrophil count (26%), decreased white blood cell count (16%), and anemia (7%). Notably, in two previous studies that treated MIBC patients with neoadjuvant ICI combined with GC regimen, the incidence of grade ≥3 absolute neutrophil count decreased or anemia occurred in 36%–40% or 10%–11% of patients, respectively.^20^, ^21^ Grade 3/4 thrombocytopenia was reported in only 5% of patients in this study, while it was reported in 34% of patients in MIBC patients treated with neoadjuvant pembrolizumab and GC regimen.^20^ Regarding non‐hematologic TRAE, only one case of grade 3 nausea was observed in this study, and no other grade 3/4 non‐hematologic AEs, other than nausea and irAE, were reported. Treatment‐related grade 3/4 hyponatremia was reported in 5%–7% of MIBC patients treated with pre‐operative ICI combined with GC chemotherapy.^20^, ^21^ The most common irAE in this study was rash, and the incidence was consistent with that in a previous study.^21^ To minimize the risk of toxicities associated with administering multiple drugs concurrently, cisplatin was given on the second day. This approach may also help decrease the occurrence of kidney function impairment and gastrointestinal reactions.

No significant correlation was found between PD‐L1 expression and pathologic response in this study or in other studies of ICI‐chemotherapy as neoadjuvant treatment for MIBC patients.^20^, ^21^ However, in studies of ICI monotherapy as neoadjuvant for MIBC patients, the results were controversial. In the pure‐01 study, pathologic response was significantly correlated with PD‐L1 expression and TMB.^32^ In the ABACUS trial, pCR rate did not show a significant difference between PD‐L1 positive and negative patients.^33^

Recent studies suggest that mutations in the DDR pathway in UC may be associated with improved responses to NAC and prolonged survival.^34^, ^35^, ^36^ It is hypothesized that these mutations may increase the sensitivity of tumor cells to cisplatin‐induced DNA damage, thereby enhancing the drug's efficacy in these tumors.^37^ The incidence of DDR gene mutations was more frequent in the pCR group (75.0%) than in the non‐pCR group (68.8%) in this study, which is consistent with findings from the PURE‐01 trial (52% vs 24%).^38^ However, no single‐gene alteration was identified as significantly different between the pCR and non‐pCR groups in both this study and the PURE‐01 trial, possibly due to the limited number of patients. This will be further explored in the second randomized stage of this study.

FRG1 has been found to play a role in both tumorigenesis and angiogenesis, and several previous studies have demonstrated an association between FRG1 expression and OS in various types of tumors.^39^ Additionally, reduced FRG1 expression has been linked to prostate cancer progression, as well as alterations in prostate cancer cell migration and invasion.^40^ TTN is a well‐known gene that encodes for myosin. However, recent evidence suggests that patients with TTN mutation have been found to have longer OS following ICI treatment in melanoma.^41^ Additionally, TTN mutation has been linked to high immunogenicity and inflammatory tumor immune microenvironment, indicating that TTN mutation may serve as a potential predictive marker for lung adenocarcinoma patients who may benefit from ICIs.^42^ Our study found that a numerically higher percentage of patients in the pCR group had TTN mutations compared to those in the non‐pCR group (67% vs. 25%). Further research is needed to confirm these findings.

There are several limitations to this study that should be considered. First, the results presented are from the single‐arm stage of the study, and therefore, further evidence from the ongoing randomized stage is required to strengthen the findings. Second, the follow‐up period may have been inadequate to capture all instances of recurrence, and longer follow‐up is needed to provide a more comprehensive understanding of the survival benefit of ICI‐chemotherapy as neoadjuvant treatment for MIBC patients. Moreover, some patients opted not to undergo RC, which may result in an overestimation of the efficacy of our analysis, as our analysis primarily focused on patients who underwent RC. Furthermore, patients who declined RC did not have available data for EFS. This issue will be addressed in the ongoing randomized controlled trial. Lastly, toxicities were only assessed for 90 days, and delayed irAEs may not have been detected within this timeframe.

## 5 CONCLUSIONS

In conclusion, neoadjuvant treatment with short‐course camrelizumab and GC regimen for MIBC patients was found to be tolerable and provided promising anti‐tumor activity.

## AUTHOR CONTRIBUTIONS

**Sujun Han:** Conceptualization (equal); data curation (equal); formal analysis (equal); investigation (equal); writing – original draft (equal); writing – review and editing (equal). **Zhigang Ji:** Investigation (equal); writing – review and editing (equal). **Junhui Jiang:** Investigation (equal); writing – review and editing (equal). **Xinrong Fan:** Investigation (equal); writing – review and editing (equal). **Qi Ma:** Investigation (equal); writing – review and editing (equal). **Linjun Hu:** Investigation (equal); writing – review and editing (equal). **Wen Zhang:** Investigation (equal); writing – review and editing (equal). **Hao ping:** Investigation (equal); writing – review and editing (equal). **Jiansong Wang:** Investigation (equal); writing – review and editing (equal). **Wanhai Xu:** Investigation (equal); writing – review and editing (equal). **Benkang Shi:** Investigation (equal); writing – review and editing (equal). **Wei Wang:** Investigation (equal); writing – review and editing (equal). **Haifeng Wang:** Investigation (equal); writing – review and editing (equal). **Honglei Wang:** Investigation (equal); writing – review and editing (equal). **Shouzhen Chen:** Investigation (equal); writing – review and editing (equal). **Hai‐Long Hu:** Investigation (equal); writing – review and editing (equal). **Jianming Guo:** Investigation (equal); writing – review and editing (equal). **Shen Zhang:** Investigation (equal); writing – review and editing (equal). **Shuai Jiang:** Investigation (equal); writing – review and editing (equal). **Quan Zhou:** Investigation (equal); writing – review and editing (equal). **Nianzeng Xing:** Conceptualization (equal); data curation (equal); funding acquisition (equal); investigation (equal); supervision (equal); writing – review and editing (equal).

## FUNDING INFORMATION

This work was supported by The Capital Health Research and Development of Special Funding (2022–1‐4021).

## CONFLICT OF INTEREST STATEMENT

The authors have no conflict of interest to declare.

## ETHICS STATEMENT

The study has been approved by institutional review board of all participant centers.

## PATIENT CONSENT STATEMENT

All patients signed inform consent to participate this study.

## PERMISSION TO REPRODUCE MATERIAL FROM OTHER SOURCES

Not applicable.

## CLINICAL TRIAL REGISTRATION

This study was registered on https://www.chictr.org.cn (ChiCTR2000032359).

## Supporting information


**Supporting information S1.** Supplementary material

## Data Availability

The data that support the findings of this study are available from the corresponding author upon reasonable request.
