# Supplementary material for: Neoadjuvant therapy with camrelizumab plus gemcitabine and cisplatin for patients with muscle‐invasive bladder cancer: A multi‐center, single‐arm, phase 2 study
Source: Cancer Med. 2023 Apr 6;12(11):12106–17. doi: 10.1002/cam4.5900 (PMC10278497; doi:10.1002/cam4.5900)

Supplementary Table 1: Treatment-related adverse events (n = 43)

| Event, n (%) | Grade 1-2 | Grade 3 | Grade 4 | Any grade |
| --- | --- | --- | --- | --- |
| Any | 26 (60.5) | 13 (30.2) | 2 (4.7) | 41 (95.3) |
| Anemia | 27 (62.8) | 3 (7.0) | 0 | 30 (69.8) |
| Nausea | 27 (62.8) | 1 (2.3) | 0 | 28 (65.1) |
| WBC decreased | 21 (48.8) | 7 (16.2) | 0 | 28 (65.1) |
| ANC decreased | 13 (30.2) | 11 (25.6) | 0 | 24 (55.8) |
| Constipation | 16 (37.2) | 0 | 0 | 16 (37.2) |
| Anorexia | 15 (34.9) | 0 | 0 | 15 (34.9) |
| ALC decreased | 12 (27.9) | 1 (2.3) | 0 | 13 (30.2) |
| Rash | 11 (25.6) | 0 | 0 | 11 (25.6) |
| Vomiting | 9 (20.9) | 0 | 0 | 9 (20.9) |
| ALT increased | 8 (18.6) | 0 | 0 | 8 (18.6) |
| Platelet count decrease | 6 (14.0) | 0 | 2 (4.7) | 8 (18.6) |
| Hypoalbuminemia | 5 (11.6) | 0 | 0 | 5 (11.6) |
| Hyponatremia | 5 (11.6) | 0 | 0 | 5 (11.6) |
| AST increased | 5 (11.6) | 0 | 0 | 5 (11.6) |
| Creatinine increased | 5 (11.6) | 0 | 0 | 5 (11.6) |
| Positive occult blood | 4 (9.3) | 0 | 0 | 4 (9.3) |
| Alkaline phosphatase increased | 4 (9.3) | 0 | 0 | 4 (9.3) |
| Hypokalemia | 3 (7.0) | 0 | 0 | 3 (7.0) |
| Hypomagnesemia | 3 (7.0) | 0 | 0 | 3 (7.0) |
| Fever | 3 (7.0) | 0 | 0 | 3 (7.0) |
| Hiccups | 3 (7.0) | 0 | 0 | 3 (7.0) |
| Dizziness | 3 (7.0) | 0 | 0 | 3 (7.0) |
| Urticaria | 3 (7.0) | 0 | 0 | 3 (7.0) |
| Hyperuricemia | 3 (7.0) | 0 | 0 | 3 (7.0) |
| Hyperglycemia | 3 (7.0) | 0 | 0 | 3 (7.0) |
| GGT increased | 2 (4.7) | 0 | 0 | 2 (4.7) |
| Upper respiratory infection | 2 (4.7) | 0 | 0 | 2 (4.7) |
| Hypochloremia | 2 (4.7) | 0 | 0 | 2 (4.7) |
| Hyperthyroidism | 2 (4.7) | 0 | 0 | 2 (4.7) |
| Hypothyroidism | 2 (4.7) | 0 | 0 | 2 (4.7) |
| Proteinuria | 2 (4.7) | 0 | 0 | 2 (4.7) |
| LDH increased | 2 (4.7) | 0 | 0 | 2 (4.7) |
| Blood glucose increased | 2 (4.7) | 0 | 0 | 2 (4.7) |
| Bone marrow suppression | 0 | 2 (4.7) | 0 | 2 (4.7) |
| Fatigue | 1 (2.3) | 0 | 0 | 1 (2.3) |
| Weight loss | 1 (2.3) | 0 | 0 | 1 (2.3) |
| Hematochezia | 1 (2.3) | 0 | 0 | 1 (2.3) |
| Prealbumin decreased | 1 (2.3) | 0 | 0 | 1 (2.3) |
| RCCEP | 1 (2.3) | 0 | 0 | 1 (2.3) |
| Cerebral infarction | 0 | 1 (2.3) | 0 | 1 (2.3) |
| Palpitations | 1 (2.3) | 0 | 0 | 1 (2.3) |
| Pruritus | 1 (2.3) | 0 | 0 | 1 (2.3) |
| WBC increased | 1 (2.3) | 0 | 0 | 1 (2.3) |
| Diabetes mellitus | 1 (2.3) | 0 | 0 | 1 (2.3) |
| Red blood cell count decreased | 1 (2.3) | 0 | 0 | 1 (2.3) |
| Abnormal liver function | 1 (2.3) | 0 | 0 | 1 (2.3) |
| Intestinal obstruction | 1 (2.3) | 0 | 0 | 1 (2.3) |
| Renal failure | 1 (2.3) | 0 | 0 | 1 (2.3) |
| Alopecia | 1 (2.3) | 0 | 0 | 1 (2.3) |
| Abdominal distension | 1 (2.3) | 0 | 0 | 1 (2.3) |
| Platelet count increased | 1 (2.3) | 0 | 0 | 1 (2.3) |
| Hematuria | 1 (2.3) | 0 | 0 | 1 (2.3) |
| Blood urea increased | 1 (2.3) | 0 | 0 | 1 (2.3) |
| Blood uric acid increased | 1 (2.3) | 0 | 0 | 1 (2.3) |
| Hemoglobin decreased | 1 (2.3) | 0 | 0 | 1 (2.3) |
| Blood bilirubin increased | 1 (2.3) | 0 | 0 | 1 (2.3) |
| Edema face | 1 (2.3) | 0 | 0 | 1 (2.3) |
| Hypertriglyceridemia | 1 (2.3) | 0 | 0 | 1 (2.3) |
| Hyperlipidemia | 1 (2.3) | 0 | 0 | 1 (2.3) |

Note: WBC, white blood cell count; ANC, absolute neutrophil count; ALC, absolute lymphocyte count; ALT, Alanine transaminase; AST, Aspartate transaminase; GGT, gamma-glutamyl transferase; LDH, lactate dehydrogenase; RCCEP, reactive cutaneous capillary endothelial proliferation.

Figure S1. Boxplot showing Tumor Mutation Burden (TMB) (A) and PD-L1 expression (B) between non-pCR and pCR patients. Difference was indicated with P values.


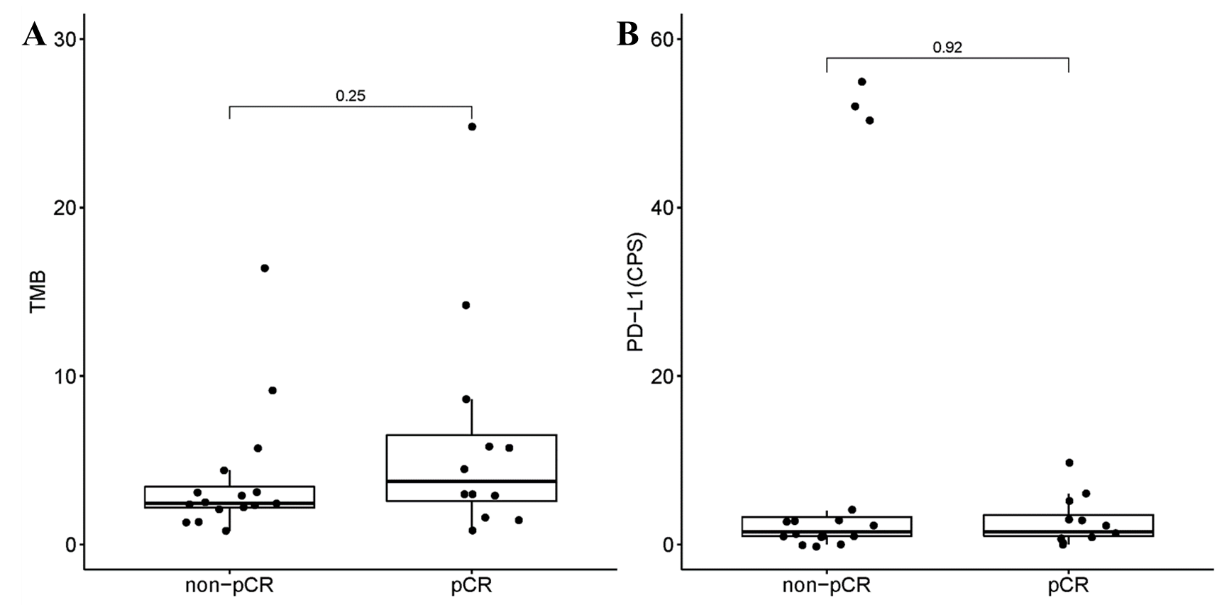


Figure S2. Genomic mutation profiles of patients with pathological complete response (pCR) and non-pCR. Top row presented the Tumor Mutation Burden (TMB) of each patient. The mutation frequency of DNA damage response and repair (DDR) genes, driver genes and somatic genes were presented in separate panels among pCR and non-pCR patients. The first row of DDR mutation panel indicated any DDR gene mutation.


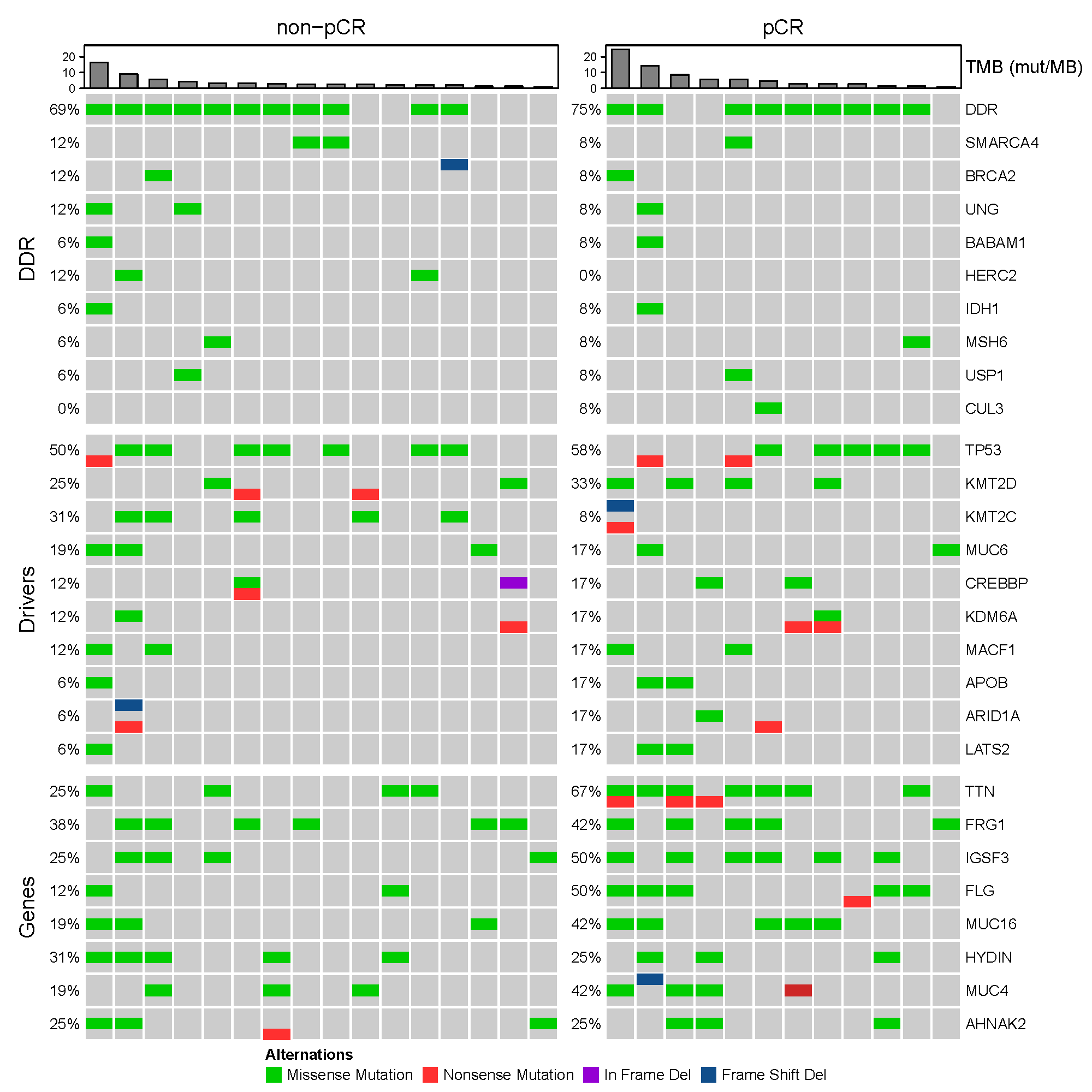

Supplement: Supplementary file 1 — Supporting information S1. Supplementary material [file CAM4-12-12106-s001.docx]
